# Recurrent graft failure secondary to portal vein steal syndrome: a case report with an unusual indication for a third liver transplant

**DOI:** 10.1186/s12893-022-01475-5

**Published:** 2022-01-22

**Authors:** Alessandro Tropea, Marco Barbàra, Duilio Pagano, Gianluca Marrone, Ioannis Petridis, Sergio Li Petri, Davide Cintorino, Salvatore Gruttadauria

**Affiliations:** 1 Department for the Treatment and Study of Abdominal Diseases and Abdominal Transplantation, IRCCS ISMETT (Mediterranean Institute for Transplantation and Advanced Specialized Therapies), Via E. Tricomi 1, 90127 Palermo, Italy; 2 Research Department, IRCCS ISMETT (Mediterranean Institute for Transplantation and Advanced Specialized Therapies), Palermo, Italy; 3 Radiology Unit, Department of Diagnostic and Therapeutic Services, IRCCS ISMETT (Mediterranean Institute for Transplantation and Advanced Specialized Therapies), Palermo, Italy; 4 Department of Surgery and Surgical and Medical Specialties, University of Catania, Catania, Italy

**Keywords:** Liver transplantation, Liver retransplantation, Portal vein anastomoses, Splenocaval shunt, Case report

## Abstract

**Background:**

Portal vein shunt is common in chronic hepatic diseases and after a liver transplant. Ensuring a satisfactory portal flow is essential to support a rapid liver recovery, of paramount importance to meet the recipient’s metabolic needs.

**Case presentation:**

We report the case of a 32-year-old female undergoing a third liver transplant due to recurrence of graft failure secondary to portosystemic shunting. The patient, affected with biliary atresia, was first transplanted in 2009 with a right split liver graft. The clinical course was complicated by biliary stenosis of the Roux-en-Y anastomosis and multiple episodes of acute rejection treated with steroid boluses, plastic dilation of the biliary anastomosis, and biliary catheter placement. Unfortunately, in 2017 a liver biopsy showed an autoimmunity with histological evidence of ANA 1:80 (granular and nucleolar pattern). This was a contributing factor of liver function impairment, leading to the need to perform a second liver transplant, complicated by an acute rejection, with only a partial response to steroid therapy. Due to the further worsening of the liver function (MELD: 40, Child–Pugh: C11), the patient was relisted for a liver transplant. After five days, she received her third liver transplant, with an entire graft of an AB0 identical group. Intraoperative exploration revealed multiple collaterals and large splenocaval shunts, with a significant alteration of the portal flow and hypertension, isolated and closed with a vascular stapler to restore the graft's regular portal vein flow.

**Conclusions:**

In patients listed for a liver transplant, portal steal syndrome should be identified prior to the transplant. Our recommendation is to consider intraoperative or perioperative closure of the portal collateral varices.

## Background

Orthotopic liver transplantation (OLTx) is frequently followed by vascular complications with a high incidence of graft loss and mortality.

Portal vein thrombosis (PVT) with complete or partial obstruction of the portal vein flow (PVF) is a known development of acute or advanced chronic liver disease and a major risk during operative and perioperative time [1].

Diagnosis and early treatment of vascular thrombosis in cirrhotic patients can improve the success rate of liver transplantation. This makes it absolutely necessary to perform regular ultrasound follow-up to evaluate the portal flow and establish a surgical program during the transplant, such as portal obstruction or vascular reconstruction with a portal by-pass.

Literature reports a PVT incidence at the time of transplant evaluation of 26% in patients requiring an OLTx [2].

Portal vein shunt is common in chronic hepatic disease and after a liver transplant. Ensuring a satisfactory portal flow is mandatory to support a rapid liver recovery, which is paramount to meet the recipient’s metabolic needs [3].

Portal hypertensive liver cirrhosis leads to a progressively-increased resistance of the hepatic sinusoids, which in turn prompts the shunting of portal flow in the systemic circulation via less resistive collateral vessels [4–7].

We report the case of a 32-year-old female at her third liver transplant due to recurrence of graft failure secondary to portosystemic shunting.

## Case presentation

The patient, affected with biliary atresia, was first transplanted in 2009 with a right split liver graft. Clinical course was complicated by biliary stenosis of the Roux-en-Y anastomosis and multiple episodes of acute rejection treated with steroid boluses, plastic dilation of the biliary anastomosis, and biliary catheter placement. Unfortunately, in 2017 a liver biopsy showed an autoimmunity with histological evidence of antinuclear antibodies (ANA) 1:80 (granular and nucleolar pattern). This was a contributing factor of liver function impairment, requiring a second liver transplant.

Due to the complete portal vein thrombosis, during the operation a venous conduit was performed with an interposition graft of cadaveric iliac vein, anastomosed between the native spleno-mesenteric vein confluence and the main portal vein of the graft.

**Fig. 1:**
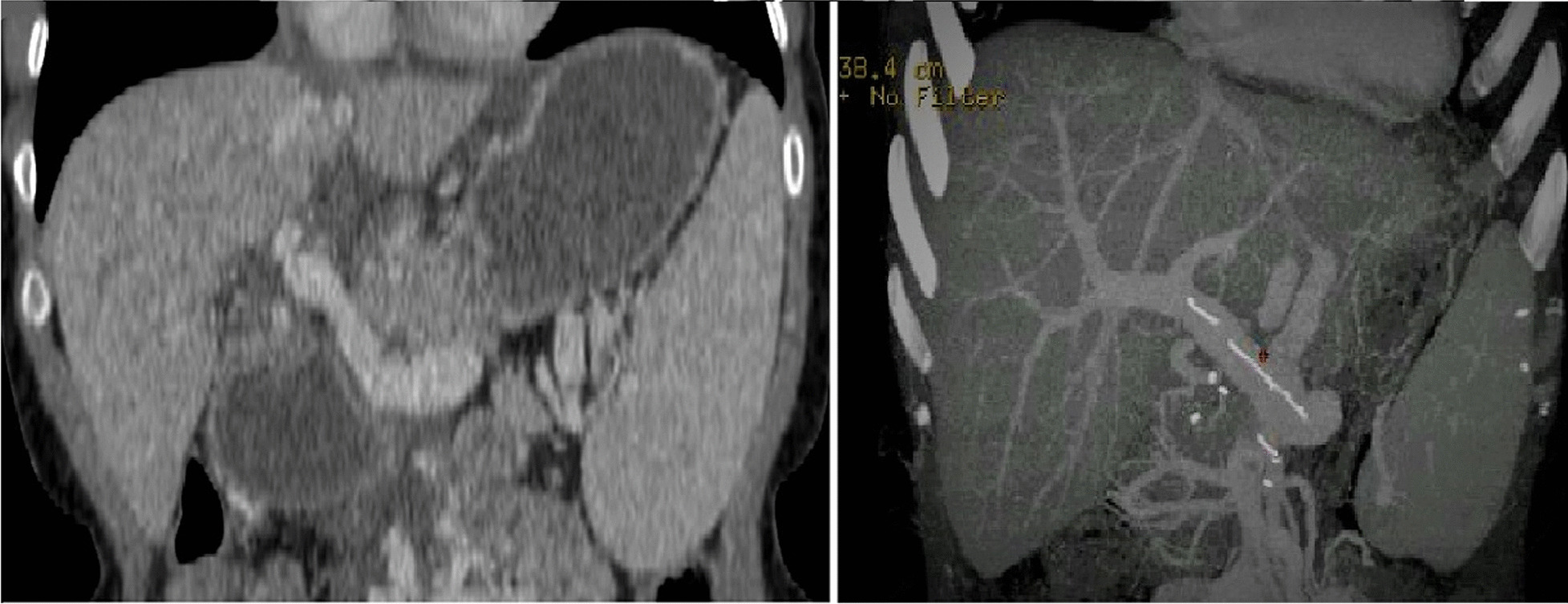
Coronal-MPR and axial-MIP images showing portal vein reconstruction with jumping graft interposition. **A**, **B** CT scan MPR images showing portal vein reconstruction with the jumping graft from the superior mesenteric vein

This procedure had a good clinical outcome, with a complete recovery three weeks after surgery. Computed tomography (CT) scan was performed with intravenous contrast and vascular reconstruction: multiplanar reconstruction (MPR), maximum intensity projection (MIP), and volume rendering (VR) showed a perfect patency of the venous conduit (Fig. 1a).

After one year, the woman became pregnant and delivered a healthy girl. In 2021, there was a new onset of jaundice and acute liver rejection. The patient underwent radiological assessment with CT scan to exclude portal vein thrombosis and related vascular occlusion. There was a tomographic finding of patent venous conduit and hypertrophy of the azygos and hemiazygos systems, and large retroperitoneal varices with wide outflow in the subrenal vena cava (Fig. 1b). Doppler ultrasound (US) was performed with severe steal of portal flow via diversion flow, exhausting the normal hepatopetal perfusion due to portal shunt. A Doppler US documented normal parameters and waveforms of the hepatic artery and portal and hepatic veins.

An acute rejection was diagnosed with a rejection activity index (RAI) of 8/9, treated with steroid bolus with partial response. Due to the further worsening of the liver function (Model for End-Stage Liver Disease (MELD): 40, Child–Pugh: C11), the patient was relisted for a liver transplant. After five days, she received a third liver transplant, with an entire graft of an AB0 identical group.

A Doppler US confirmed an inadequate portal inflow of the graft, and the need to suture the wide varices to restore the portal inflow.

Intraoperative exploration revealed multiple collaterals and large splenocaval shunts, with a significant alteration of the portal flow and hypertension, isolated and closed with a vascular stapler to restore the graft's regular portal vein flow.

The recipient’s portal vein was anastomosed in an end-to-end fashion between the old venous conduit and the graft portal vein.

The stapler transection was immediately followed by an improvement of the portal vein flow, evidenced on Doppler (81.4 cm/s). Conventional arterial reconstruction was performed between the recipient's arterial and the graft's hepatic artery. Wider Roux-en-Y biliary reconstruction was performed.

**Fig. 2:**
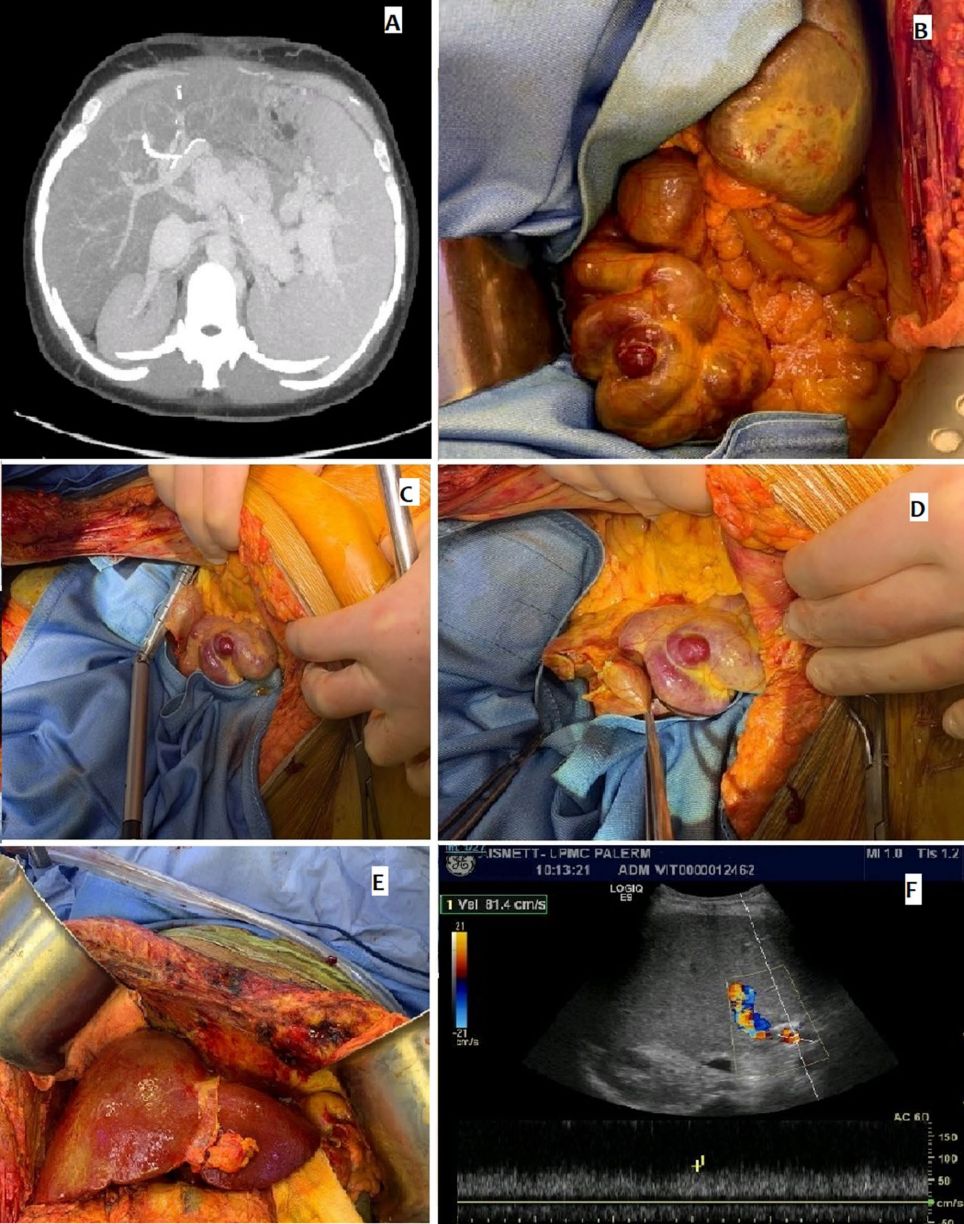
Intraoperative findings—giant spleno-renal shunt: **A** Tomographic image of splenocaval shunt; **B** Intraoperative findings of giant splenocaval shunt; **C**, **D** Stapler resection of the shunt; **E** Third liver after reperfusion; **F** Postoperative Doppler US

A routine Doppler US after the transplant confirmed an adequate portal blood flow (Fig. 2), and the patient was discharged 15 days after transplantation.

## Discussion and conclusions

A portosystemic shunt (PSS) is an abnormal vascular connection between the portal vein branches and the systemic circulation in the liver. The natural history of a PSS is strongly dependant on its gauge. The shunt will tend to progressively increase, often causing an inversion of the flow and the related clinical symptoms. Portal flow variations can cause occlusion or PVT, one of the most feared complications during a transplant.

The aim of this report is also to highlight the importance of a preoperative study of transplant candidates with a personalized surgical program for patients with PVT. These patients require an accurate Doppler US to highlight potential alterations in the normal vein waveform, which must always be investigated with a CT scan to evaluate the portal vein integrity or the presence of thrombosis.

Progressive PSS can determine graft failure through a stealing of portal flow due to changes in portal venous anatomical system and in the hemodynamics. Patients affected by PSS always develop systemic syndromes, such as ascites and hepatorenal syndrome, which can cause a worsening of the renal function leading to renal insufficiency with considerable sodium retention. Progressive peripheral vasodilation increases the glomerular filtration rate determining a renal accumulation of angiotensin II. The increased tubular sodium reabsorption generates an ascitic decompensation that initially appears responsive to depletion diuretic treatment, but that often has a degenerative trend developing refractory ascites that requires paracentesis.

At this regard, a portosystemic shunt must be closed to avoid inadequate portal inflow to the graft, leading to graft loss. Increased hepatic vascular resistance due to acute rejection, fluids overload, or a small-for-size graft can hinder spontaneous regression of the shunt.

Accurate tomographic vascular reconstruction is necessary [8–10] to assess:

1. site and size of varices;
2. diameter and patency of the main portal vein in order to establish a clinical strategy.

At present, there are no shared guidelines for the management of portosystemic shunt, before or after liver transplantation, and evidence of effective treatments is indeed limited.

After a multidisciplinary evaluation and review of the clinical reports, we identified the occurrence of portal vein shunt as the cause of progressive graft impairment in our patient.

In the case reviewed, we found strong evidence of the importance of portosystemic steal in causing chronic liver damage without thrombosis of the main portal vein [11, 12].

In our tomographic reconstruction, before the third transplant, we were able to appreciate a normal portal vein, with no signs of thrombosis and ultra-sonographic evidence of hepatofugal flow.

In patients listed for a liver transplant, portal steal syndrome should be identified prior to transplant, and we also recommend to consider intraoperative or perioperative closure of the portal collateral varices.

## Data Availability

The datasets used and/or analysed during the current study are available from the corresponding author on reasonable request.

## Abbreviations

OLTx: Orthotopic liver transplantatio
PVT: Portal vein thrombosis
PVF: Portal vein flow
ANA: Antinuclear antibodies
CT: Computed tomography
MPR: Multiplanar reconstruction
MIP: Maximum intensity projection
VR: Volume rendering
US: Ultrasound
RAI: Rejection activity index
MELD: Model for End-Stage Liver Disease
PSS: Portosystemic shunt
